# COVID-19 infection initially presented by interstitial edematous pancreatitis and acute peri-pancreatic collection; the first CT case report in Egypt

**DOI:** 10.1186/s43055-021-00611-0

**Published:** 2021-09-20

**Authors:** Ahmed Samir, Abdelaziz Elnekeidy, Enjy El-Kady

**Affiliations:** Department of Radio-Diagnosis, Faculty of Medicine, Alexandria University, 17 Champollion Street, El-Messalah, Al-Kartoom Square, Al-Azareta, Alexandria, 21526 Egypt

**Keywords:** COVID-19, Pancreatitis, Case report

## Abstract

**Background:**

The extra-pulmonary computed tomography (CT) findings of severe acute respiratory syndrome coronavirus 2 (SARS-CoV-2) or coronavirus disease 2019 (COVID-19) have been described in the literature, including the neurologic, cardiac, and abdominal manifestations. Ischemic and inflammatory bowel changes were the most frequent abdominal CT findings. Acute pancreatitis was seldom found all over the world outside of China and described in case reports, but not in Egypt.

**Case presentation:**

A 44-years-old female patient presented with severe abdominal pain, vomiting, and mild fever for six days. No respiratory symptoms were encountered till the time of the radiological investigation. Abdominal CT examination revealed interstitial edematous pancreatitis with acute peri-pancreatic fluid collection according to the revised Atlanta classification. No pancreatic necrosis or vascular complications were depicted. Secondary pyloro-duodenitis was noticed. The modified Balthazar CT-severity index was moderate (6/10). Basal chest scans showed bilateral variable-sized bronchocentric and sub-pleural consolidative pneumonic patches with mild bilateral pleural effusion. The patient was admitted to the intensive-care unit (ICU) for two weeks. The serum amylase and lipase titers were elevated and the polymerase chain reaction (PCR) test for COVID-19 was positive. She received pancreatic, circulatory, and pulmonary medical support, then she was discharged after stabilization of her condition.

**Conclusion:**

The authors provided this case report for the association between COVID-19 infection and acute pancreatitis, which is mostly the first in Egypt. It documented their Egyptian experience to be added to the international literature. It radiologically described their chest and abdominal CT findings in detail using the COVID-19 Reporting and Data System (CO-RADS) and the revised Atlanta classification with modified Balthazar CT-severity index (CTSI). This could eventually enrich the radiological point of view in addition to the previously published clinical case reports.

## Background

Several extra-pulmonary manifestations of SARS-CoV-2 (COVID-19) have been described, including the neurologic, cardiac, and abdominal manifestations [1]. The ischemic bowel changes were the most common abdominal complication accompanying COVID-19 [2]. Acute pancreatitis is one of the rare complications of COVID-19 infection which was seldom found all over the world outside of China, but not in Egypt. It was described in some case reports by gastroenterologists and surgeons, focusing mainly on the clinical scenario in addition to some radiological data [3–6]. On the other hand, very few case reports were offered by radiologists and detailed the radiological changes of the disease [7, 8].

Scientists attributed the pancreatic injury that accompanies COVID-19 infection to either primary direct viral attack or secondary angiotensin receptor-converting-enzyme 2 abnormalities by the viral proteins [9]. Consequently, acinar and ductal damage can occur in addition to islet cell damage and ischemic changes [3]. The exclusion of other causes of pancreatitis such as cholelithiasis and the confirmation of COVID-19 infection is essential in such cases [3].

The authors provide this case report for the association between COVID-19 infection and acute pancreatitis, which is mostly the first in Egypt.

Different from the majority of the previous case reports in the literature, acute pancreatitis in the current case report was the initial clinical presentation that preceded the respiratory symptoms of COVID-19 infection. The authors hence believed that COVID-19 infection should be always concerned in patients presenting with acute pancreatitis without signs of cholelithiasis or a history of metabolic diseases or alcohol consumption.

Additionally, the authors were keen to radiologically describe their chest and abdominal CT findings in detail using the CO-RADS and the revised Atlanta classification with modified Balthazar CT-severity index (CTSI).

## Case presentation

A 44-years-old Egyptian female patient presented to our radiology department in June 2021 with a clinical request for abdominal CT imaging. The referring clinician suspected pancreatitis after performing an ultrasound examination for the patient in the out-patient clinic.

*Clinically,* the patient was complaining of acute abdominal pain and vomiting for six days. The patient was feverish at 38 degrees celsius (38 °C). She did not have any respiratory complaints till the date of the radiological investigation. The past history was negative regarding diabetes mellitus, hyperlipidemia, hypertension, and auto-immune diseases. She had a surgical history of cholecystectomy five years ago.

*Radiologically, the abdominal scans showed:*

- A large peri-pancreatic and left anterior pararenal homogenous and hypo-attenuated fluid collections measuring 8 Hounsfield units (8 Hu) with a mild stranding of the nearby fat planes. It extends to the lesser sac with nearby mural thickening and sub-mucosal edematous changes of the pyloro-duodenal junction, denoting secondary gastro-duodenitis.
- The pancreas itself retained a normal size and homogenous tissue matrix without non-enhancing necrotic areas. The main pancreatic duct (MPD) was not dilated.
- The superior mesenteric vessels as well as the portal vein, superior mesenteric vein, and splenic vein were patent and adequately opacified without vascular thrombosis.
- The spleen was mildly enlarged, reaching 14 × 10.6 × 6 cm in maximum cranio-caudal (CC), anteroposterior (AP), and side to side (at the level of splenic hilum) dimensions (with 542 ml splenic volume and 882 splenic index). It showed a normal homogenous tissue matrix and absent focal lesions.
- No ascites were detected elsewhere. Clear cholecystectomy surgical bed with an average caliber of the common bile duct (CBD) and intrahepatic biliary radicles.
- Mild bilateral pleural collection was noted.
- The fore-mentioned CT findings were suggestive of interstitial edematous pancreatitis (IEP) with acute peri-pancreatic fluid collection (APFC) according to the revised Atlanta Classification. The modified Balthazar CT-severity index (CTSI) was 6/10 (moderate).

**Fig. 1:**
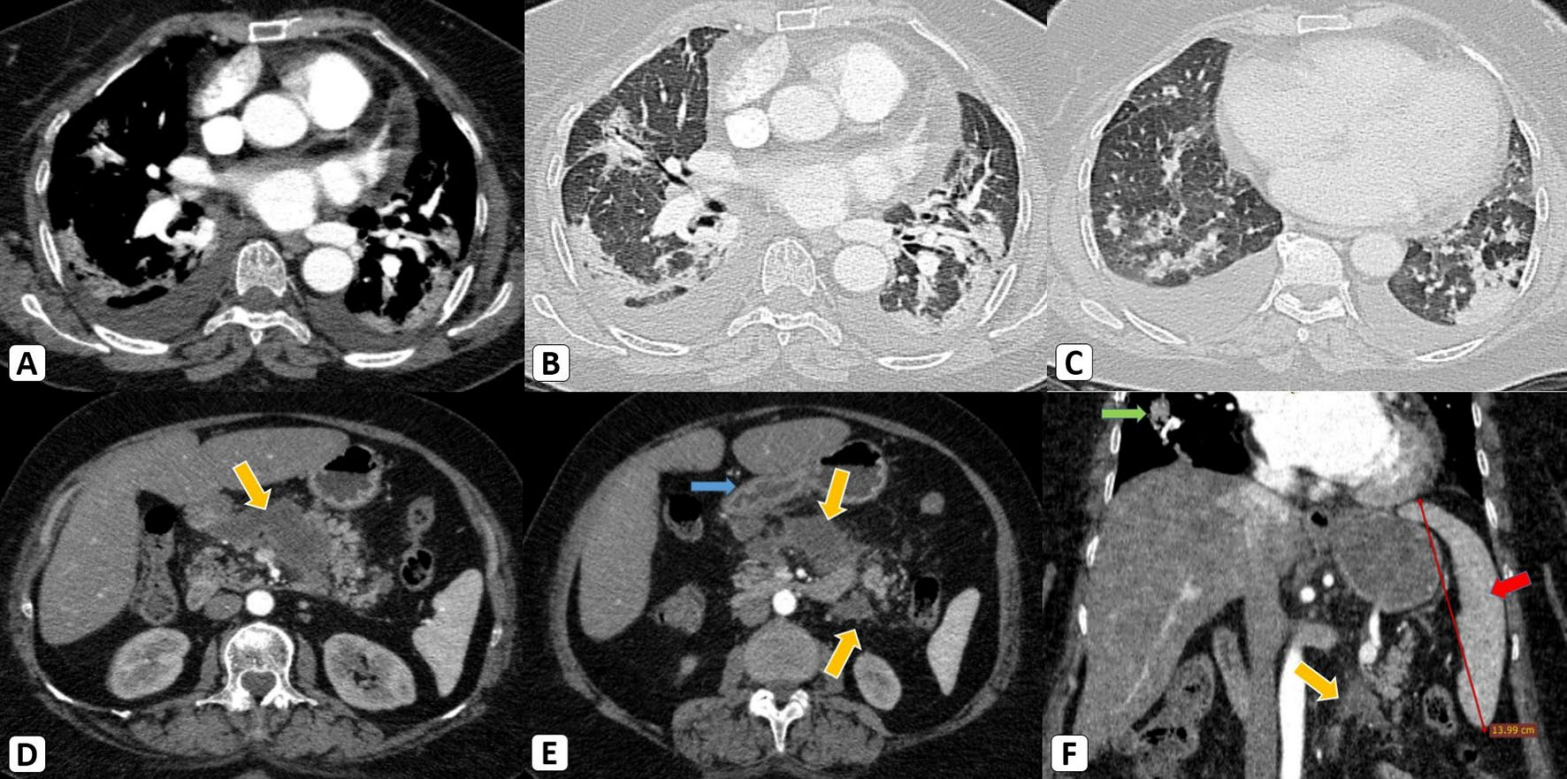
**A**–**C** Basal chest CT cuts (mediastinal and lung window) showed bilateral variable-sized bronchocentric and sub-pleural consolidative pneumonic patches (CO-RADS 5) with a mild bilateral pleural collection. **D**–**E** Axial abdominal CT cuts revealed a large peri-pancreatic and left anterior para-renal homogenous and hypo-attenuated fluid collections (8 Hu) with mild stranding of the nearby fat planes (yellow arrows). Nearby mural thickening and sub-mucosal edematous changes of the pyloro-duodenal junction, denoting secondary gastro-duodenitis (blue arrow). The pancreas retained homogenous parenchyma without necrotic areas. The main pancreatic duct (MPD) was not dilated. [F] Coronal abdominal CT cut revealed mildly enlarged spleen, reaching 14 cm in maximum bi-polar diameter, without focal lesions. The pancreatic collection (yellow arrow) and basal pneumonic changes (green arrow) are obvious *… COVID-19 infection complicated with acute pancreatitis and an acute peri-pancreatic fluid collection was the diagnosis according to the revised Atlanta Classification. The Balthazar modified CT-severity index (CTSI) was 6/10 (moderate)*

*The basal chest CT cuts* additionally showed bilateral variable-sized bronchocentric and sub-pleural consolidative patches, they were suggestive of basal pneumonic changes. The viral origin was highly suspicious radiologically (CO-RADS 5), hence clinico-laboratory correlation was requested. This was attributed to the current pandemic status, in addition to the multi-centricity and characteristics appearance of the pulmonary parenchymal involvement. (Fig. 1).

The patient was urgently admitted to the intensive care unit (ICU). At the time of ICU admission, the patient had mild tachypnea (20 breaths/min) and mild tachycardia (92 beats/min). His blood pressure was 130/80. The serum amylase and lipase titers were significantly increased (672 and 1619 U/L respectively) and this confirmed the diagnosis of acute pancreatitis. The PCR test for COVID-19 was performed and proved positive. The white cell count was not elevated (9.5 × 10^9^/L). The C-reactive protein (CRP) was elevated at 77.5 mg/L. The serum ferritin and D-dimer were elevated also (338 and 443 ng/mL respectively). The vital signs and laboratory tests results of the patients are detailed in (Table 1).

**Table 1:** The vital signs and initial lab investigations at the time of ICU admission

Vital signs	
Temperature	38 °C
BP	130/80
Respiratory rate	20 breaths/min
Heart rate	92 beats/min
O_2_ Sat at room air	90%
CBC	
HB	10.7 g/dl
HT	33.0%
WBC	9.5 × 10^9^/L
PLT	170 × 10^9^/L
PT/activity	12.1 s/84.1%
INR	1.08
PTT	39.1 s
Na	136 mEq/L
K	4.5 mEq/L
CRP	77.5 mg/L (high)
Serum ferritin (ng/mL)	338 ng/mL (high)
D-dimer (ng/mL)	443 ng/mL (high)
Bilirubin (total/direct)	0.31/0.15 mg/dL
Rena function tests	
Urea	97 mg/dL
Creatinine	5.34 mg/dL
Cardiac enzymes	
CKMB	2.5 U/L (average)
Troponin	0.2 ng/mL (average)
Pancreatic enzymes	
Serum lipase	1619 U/L (high)
Serum amylase	672 U/L (high)
Tumor markers	
Alpha-fetoprotein	1.53 ng/ml (negative)
Serum CEA	1.55 µg/L (negative)
CA 19.9	7.13 units/ml (negative)
RT-PCR for SARS-CoV-2	Tested positive

The patient stayed in the intensive care unit (ICU) for two weeks and received pancreatic and pulmonary medical support. This included off-oral intake with IV-fluids replacement, high-flow nasal oxygen, prophylactic heparin anticoagulation, intravenous prophylactic antibiotics, and dexamethasone. She was discharged later after stabilization of her condition. The fever had subsided. The leukocytic count decreased (5.3 × 10^9^/L). The C-reactive protein (CRP) also decreased (24 mg/L). The serum amylase and lipase titers had significantly decreased (77 and 34 U/L respectively). The renal functions had improved.

## Discussion

Acute pancreatitis can be induced by several etiologies in adults. The most common cause is cholelithiasis, followed by alcohol abuse and auto-immune diseases as well as metabolic diseases. Additionally, the relationship between viral infections and acute pancreatitis had been studied, including viruses such as Epstein–Barr virus, measles, mumps, Coxsackie B, and hepatitis viruses [10]. Furthermore, influenza A virus subtype H1N1 had also been correlated with acute pancreatitis in some reports [11]. Shortly after the announcement of the current pandemic, the relation between COVID-19 and acute pancreatitis was investigated [12]. Since the extra-pulmonary complications of COVID-19 are currently well-known, therefore, every radiologist should pay attention to these associations [8].

In this case report, exclusion of other causes of pancreatitis was established clinically and radiologically and COVID-19 infection was proved using PCR-test. The diagnosis of acute pancreatitis preceded that of COVID-19 infection because of the initial absence of respiratory symptoms. This matched the previous case reports provided by Kandasamy et al. [5], Karimzadeh et al. [13] and Wang et al. [14]. On the other hand, this was different from the previous case reports provided by Al Mazrouei et al. [7], Mohammadi Arbati et al. [15], Anand et al. [16], Gupta et al. [17], and Lakshmanan et al. [18], where the COVID-19 infection preceded the diagnosis of acute pancreatitis.

This case report was diagnosed as interstitial edematous pancreatitis with acute peri-pancreatic fluid collection according to the revised Atlanta classification. This was similar to the previous case reports provided by Kandasamy et al. [5], Al Mazrouei et al. [7], Wang et al. [12], Gupta et al. [17], and Lakshmanan et al. [18]. On the other hand, it was different from the previous rare case report provided by Mohammadi Arbati et al. [15] which described an acute necrotizing form of pancreatitis with COVID-19 infection.

## Conclusion

The authors provided this case report for the association between COVID-19 infection and acute pancreatitis, which is mostly the first in Egypt. It documented their Egyptian experience to be added to the international literature. This could eventually enrich the radiological point of view in addition to the previously published clinical case reports. Acute interstitial edematous pancreatitis with acute peri-pancreatic fluid collection was the initial clinical presentation that even preceded the respiratory symptoms and signs of COVID-19 infection. Chest and abdominal CT findings were detailed using the CO-RADS and the revised Atlanta classification with modified Balthazar CT-severity index (CTSI). These CT findings predominantly included the presence of peripheral located mixed ground glass and consolidative patches in both lung fields in addition to the bulky pancreas with stranding of the surrounding fat planes and multi-locular peri-pancreatic fluid collections.

## Data Availability

The data and material are available from the corresponding author on reasonable request.

## Abbreviations

SARS-CoV-2: Severe acute respiratory syndrome coronavirus 2
COVID-19: Coronavirus disease 2019
CO-RADS: Coronavirus disease 2019 (COVID-19) Reporting and Data System
CTSI: CT-severity index
CT: Computed tomography
PCR: Polymerase chain reaction
